# Videos engaging in conspiracy theories: Promoting or refuting foreign-pseudohistory on the video-sharing website (Bilibili)

**DOI:** 10.1371/journal.pone.0318986

**Published:** 2025-02-18

**Authors:** Yi Zhu, Yichao Wang, Siyuan Ma

**Affiliations:** 1 Department of Communication, Rollins College, Winter Park, Florida, United States of America; 2 Department of Communication, Michigan State University, East Lansing, Michigan, United States of America; 3 Department of Communication, University of Macau, Macau SAR, China; Universiti Malaya, MALAYSIA

## Abstract

Foreign pseudohistory, as one format of conspiracy theories, is an unverified discourse explicitly stating that a culture, civilization, or achievement outside one’s homeland country did not exist, does not have historical continuity, or was plagiarized from this homeland country. The current study analyzed 302 videos about foreign pseudohistory from a popular Chinese video-sharing website (Bilibili) and found 213 videos supporting foreign pseudohistory and 89 videos opposing foreign pseudohistory. Videos opposing foreign pseudohistory attract more viewers and comments than videos supporting foreign pseudohistory, but the latter videos are posted by a smaller group of core creators and also attract considerable numbers of views. The inductive thematic analysis identified three major themes from these videos, including: 1) how foreign civilizations and history were based on fabrication and plagiarism; 2) promoting foreign pseudohistory as a way to fight against Western-centrism; and 3) refuting and mocking foreign pseudohistory and enlightening the public about the real history. The implications of this study were discussed

The widespread conspiracy theories in the current media environment, especially about culture and history, are big challenges to the general public [1,2]. Scholars often need to put extra effort into refuting conspiracy theories related to pyramids, aliens, and the Atlantis kingdom [2–4], so that they would not contaminate people’s correct understanding of real history and culture. Previous literature has mainly focused on politics-related conspiracy theories in developed countries and regions [5,6] and paid much less attention to conspiracy theories of history and culture among developing countries and regions. Even though some studies mentioned developing countries’ conspiracy theories, the studies tended to attribute such a phenomenon to the rise of nationalism or the lack of education in these countries [7,8]. However, such explanations might merely touch the surface of conspiracy theories in developing countries.

Conspiracy theories about history and civilizations are important topics in developing countries and may contain a variety of subtly different themes. Meanwhile, the *existence* of conspiracy theories does not mean that conspiracy theories are *dominating* public opinion in developing countries. Academic communities also actively refute conspiracy theories and take serious reflections on them, pointing out that those pieces of false information would mislead people’s attitudes or bias their decision-making behaviors [9,10]. Overall, previous scholarship on conspiracy theories paid limited attention to developing countries [5,6], and there is a lack of understanding of how conspiracy theories about history and civilizations are invented, spread, and debated among developing countries [7,8]—even though they strongly influence people’s attitudes and behaviors in these countries.

China, as one of the biggest developing countries, is also bothered by conspiracy theories about culture and history. Many conspiracy theories have manifested themselves in the past two decades from foreign pseudohistory [11,12]. In the 2010s, Chinese scholars such as Huang Heqing and He Xin argued that foreign history outside China was overestimated in length, and many foreign achievements were copied from China [11,12]. Although their declarations were criticized by academia as extremely lacking evidence [13], the idea of foreign pseudohistory seems to be resurging and leading to many debates in the current social media age [14]. For example, we have observed thousands of foreign pseudohistory videos posted on Bilibili (one of the mainstream video platforms in China, similar to YouTube) with millions of views since 2021, and the numbers are still increasing.

How did those videos specifically explain themselves? From the video creators’ perspective, between supporters and opponents of foreign pseudohistory, which one got more audience attention? These questions are worth exploring with our current study to extend the current knowledge about the formation and communication of such discourses on social media.

## Literature review

### Conspiracy theory

Conspiracy theories refer to explanations of events or circumstances that cite as a main causal factor a small group of powerful persons, the conspirators, acting in secret for their own benefit and against the common good [15]. Conspiracy theories offer easy explanations for new phenomena threatening the original belief system [16]. Because conspiracy theories tend to promote information through emotional appeals, unsupported allegations, and unverified speculations [17], they may cause negative social outcomes such as spreading false information, making irrational decisions, and causing panics among the general public [6,17]. For instance, conspiracy theories tend to take counter-epistemic positions and consider organized science corrupt [18–20]. In addition, people with a conspiracist worldview believe that large networks of people with malevolent agendas try to secretly execute plots and mass hoaxes [16,18,21,22]. Furthermore, climate change is perceived as a hoax perpetrated by scientists to seek funding or to act out on a secret agenda by the believers of conspiracy theories [18]. The COVID-19 pandemic, according to the conspiracy theories posted on 8kun and Gab, is an anti-Trump plot or part of a mass vaccination plot by big pharma to make money [20].

According to conspiracy theory believers (conspiracists), a small group of powerful persons (conspirators) is controlling the world [15]. The conspirators can be foreign or local governments, fraternal organizations, or any other group perceived as powerful and treacherous [15,23]. However, previous studies have focused more on conspiracy theories that relate to the government and political issues rather than culture- or history-related conspiracy theories. For instance, the strong beliefs in the end of the world in the Mayan civilization, aliens in ancient times, and astrology have been considered problematic but not severe in the current postmodern society [24].

Previous studies have summarized three major motivations for engaging in conspiracy theories, including: 1) epistemic needs, such as curiosity satisfaction or uncertainty avoidance; 2) existential needs, such as restoring the sense of security and control; and 3) social needs, such as holding positive regard for one’s groups [16,25]. Their studies also propose that many people may regard conspiracy theories as entertainment or a tool for anti-hegemony [26,27] and do not need to really believe conspiracy theories. Some people discuss or spread conspiracy theories just because they think these arguments are interesting but do not care about the truth [26]. Some people treat conspiracy theories more seriously. They tend to have specific worldviews and political beliefs, such as anti-authority or living at the fringe of society [15,28]. However, these people may regard the conspiracy theories as a tool of anti-hegemony without presuming trust or distrust toward any authority. Considering the poor records of non-transparent politics and the reproducibility crisis in academia, it is a stigma to assert that people who engage in conspiracy theories are always irrational [27]. Furthermore, some scholars have also argued that public discourses on conspiracy theories show non-traditional civic engagement and participation to restore the public’s voices [19].

Different from previous studies mentioned above, some other studies also propose that culture- or history-related conspiracy theories deserve more research because they can strongly influence the whole society’s perception of important issues such as cultural and national identities [7,29,30]. Particularly, the influence of culture- or history-related conspiracy theories is considerable among developing countries [7,30].

However, researchers have limited understanding of the motivations implications for engaging in discussion about culture- and history-related conspiracy theories. Little is known about what factors motivate those who promoted culture- and history-related conspiracy theories and engage in discussion about such conspiracies. Therefore, the current study selected a certain type of culture- or history-related conspiracy theory, *pseudohistory*, to analyze how people engage in conspiracy theories.

### Pseudohistory

Pseudohistory, or weird history, is a unique form of conspiracy theory. Pseudohistory mimics the presentation of professional history but contains either unprovable evidence or unreasonable logical inferences [7]. Some pseudohistory writers have had a considerable impact around the world. For instance, the famous Russian mathematician Fomenko claimed that the length of human history since the Neolithic Age has been overly estimated [7]. Fomenko and his colleagues also reprised Slavophile historiography for their own purposes, such as claiming that all the great ancient peoples and elites, from the Etruscans to the Hittites, from Attila to Genghis Khan, were of Slavic descent [7,29,30]. Another example is some Korean writers’ work on the early civilization activities of the Korean Kingdoms, which also contained some conspiracy theories mixing myths and legends with historical records [31]. In these works, the writers traced the history of the Korean Kingdom back to 2300 BC without archaeological and documentary evidence and mistakenly regarded the emperor in the legend who lived for 2,000 years as a real person [31].

Pseudohistory is often recognized as a long-existing but not serious problem of postmodern society [24,32]. Even in developed countries with admirable higher education systems, many people still believe the stories of Mayans, Atlantis, exorcism, or channeling with ghosts [24,32]. However, the growth and popularity of pseudohistory in developing countries may benefit from more complicated social-psychological factors. Pseudohistory arguments cater to people’s curiosity and nationalistic sentiments, thus obtaining a better chance to disseminate to the general public [7]. In addition, distrust towards intellectuals and dissatisfaction with standardized academic expression could also lead to pseudohistory arguments [17,20,33]. Unlike some previous studies in developed countries, people from developing countries may face negative consequences if they underestimate the influence of pseudohistory, such as the stigmatization of specific people and biased decision-making [17,20].

China, as one of the developing countries, can be used as a typical example for studying culture- and history-related conspiracy theories, such as pseudohistory. This idea of pseudohistory can be traced back to He Xin and Huang Heqing’s works, which started in the 2010s. For example, foreign pseudohistory believers in China claim that the history of ancient Greece was invented by later generations, and many achievements during the Enlightenment and Industrial Revolution were plagiarized from China [11,12]. Therefore, the current study defines the claim of foreign pseudohistory as an unverified statement explicitly stating that a culture, civilization, or achievement outside China did not exist, does not have historical continuity, or was plagiarized from China.

### Research questions

#### Debates around pseudohistory.

Previous studies examined conspiracy-theory videos on video-sharing websites such as YouTube to investigate the themes, positions, dissemination, and effects of conspiracy theories [2,34–37]. Most of these studies used content analysis to classify YouTube conspiracy videos [34,37]. Others used qualitative analysis [35], corpus analysis of keywords [34], network analysis [4], and experiment design [36]. Nearly all of these studies focused on conspiracy videos on YouTube. Following this line of research, the current study intends to explore videos of foreign pseudohistory on Chinese video-sharing platforms. Chinese society does not overwhelmingly lean towards foreign pseudohistory but contains many debates about whether people should support or criticize such pseudohistory. Hence, the current study intends to examine the positions of videos regarding foreign pseudohistory as well as their relative popularity. The first two research questions are thus proposed as below; the current study uses quantitative methods and both descriptive and inferential statistics to answer them.

RQ1: What are the positions of videos related to foreign pseudohistory? Are there more videos supporting pseudohistory than videos criticizing pseudohistory, or vice versa?
RQ2: Comparing videos supporting foreign pseudohistory and videos criticizing foreign pseudohistory, which type of videos is more popular with the audience?

#### Motivations of engaging in pseudohistory.

Similar to other conspiracy theories, foreign pseudohistory results from multiple motivations. For example, people’s limited knowledge could trigger the acceptance of foreign pseudohistory [24], and nationalism in developing countries could sometimes exaggerate the historical achievements of their own nation [7,31]. The distrust towards intellectuals and the dissatisfaction with standardized academic expression (“official science” in their words) could also lead to pseudohistory arguments [17]. In addition, when video creators find specific content (e.g., pseudohistory) that may draw attention from a sizable audience, they may be encouraged to create more foreign pseudohistory videos for economic reasons [38].

Meanwhile, many video creators and scholars also criticize foreign pseudohistory. Their criticism targets the fallacies and unverified evidence in the pseudohistory arguments. For example, the Soviet Union’s national academy was confused and shocked by Fomenko’s pseudohistory findings [30], and China’s and South Korea’s national-level academic institutions also clearly expressed that the foreign pseudohistory arguments did not have supporting evidence [10,13].

Therefore, videos related to foreign pseudohistory involve various themes. The current study is interested in examining the themes of videos about foreign pseudohistory and proposes the third research question:

RQ3: What types of themes can be identified in foreign pseudohistory videos?

## Method

The current study first adopted both content and thematic analyses to examine the content of foreign pseudohistory videos on a Chinese video-sharing website called Bilibili. Then, metadata (release date, creator, title, abstract, comments under videos, and bullet/time-sync comments) of videos supporting and opposing foreign pseudohistory were examined.

### User demographics of Bilibili

Bilibili is one of the most popular Chinese video-sharing websites, with 96.5 million daily active users [39]. According to Statista [40,41], most of the users of this website were youth living in relatively developed urban areas. Furthermore, China Internet Watch [42] reported that approximately half of Bilibili users had bachelor’s degrees, indicating a relatively educated user demographic. In light of this, it’s expected that users of Bilibili have better judgment on conspiracy theories than the general population in China.

### Procedure of data collection and analysis

The data collected from the Bilibili website are all publicly accessible by searching the keywords; no need for the API or any other private access paths. The collected data are available in the supplementary file.

To obtain the data for the current study, one of the authors searched for the Chinese term “伪 (‘pseudo’ or ‘fake’) 史 (‘history’)” using the built-in search engine in Bilibili and scraped all results sorted by view counts (from highest to lowest). The keyword “foreign” was not used in the query, as 1) in the Chinese media context, the term “伪史” almost exclusively refers to foreign pseudohistory, and 2) it was desirable to maximize the recall rate of the search results. Videos irrelevant to the research interests were excluded from further analyses (explained subsequently). Web caches were created on September 2^nd^, 2023, prior to the data scraping process, to ensure the replicability of results, if any.

The search engine in Bilibili came with a limitation that it could only return 1,000 records; therefore, it was technically unfeasible to get the population data or its size. Instead, in the current study, only videos that were viewed more than 10,000 times were included, as they were more influential and thus had higher value to study. As a result, 383 videos were scraped.

The authors then conducted a content analysis to code videos based on the positions they held. All three authors discussed and coded the first 21 videos on the list to form a codebook with four categories: a) 1 =  support the claim of foreign pseudohistory, b) -1 =  oppose the claim of foreign pseudohistory, c) 0 =  no specific preference or mentioned both sides of the positions, and d) 99 =  completely irrelevant. Coding was based on titles, abstracts, and video content, where other metadata, including comments, were not considered. The following 122 videos were then coded by three authors separately, and a relatively high intercoder reliability was obtained (Krippendorff’s α for nominal data =  0.841). Given the high intercoder reliability, researchers coded the remaining videos in three pairs (e.g., two coders coded a single video), where Krippendorff’s α’s were equal to 0.956, 0.924, and 0.844 respectively. Discrepancies were discussed together among the three coders in order to reconcile them.

In order to further the understanding of foreign pseudohistory videos, a thematic analysis was also conducted to identify potential themes promoted in these videos. Thematic analysis is a data analytic process relying on interpretative approaches to immerse oneself in the qualitative data to identify themes [43–45]. Given the lack of studies and theories examining foreign pseudohistory videos, thematic analysis was selected to explore qualitative data to generate unanticipated insights, provide detailed features of a large body of data to answer the investigators’ research questions, and offer accessible results to both researchers and the educated general public [43] (see Braun & Clarke for details about advantages of thematic analysis).

To answer the third research question, all three authors watched all 383 videos and purposefully selected 35 videos for thematic analysis. These videos were purposefully selected because their content was most representative of either supporting or opposing the claims of pseudohistory. The authors first followed the procedure of thematic analysis proposed by Braun and Clarke [43] to identify themes from these 35 videos. Then, the authors examined specific examples and themes, made comparisons between cases, and checked if the analysis met saturation, where new themes could not be generated, as suggested by Shank [46].

## Results

### Positions of videos

To answer the first research question about the positions of videos related to foreign pseudohistory, the current study conducted a series of descriptive analyses. We first categorized videos based on their positions and relevance to foreign pseudohistory, which are presented in Table 1.

**Table 1:** Distribution of Codes.

Code	Meaning	Count
1	Support the claim of foreign pseudohistory	213
−1	Oppose the claim of foreign pseudohistory	89
0	No specific preference or mentioned both side	7
99	Completely irrelevant	74
Overall		383

**Fig 1:**
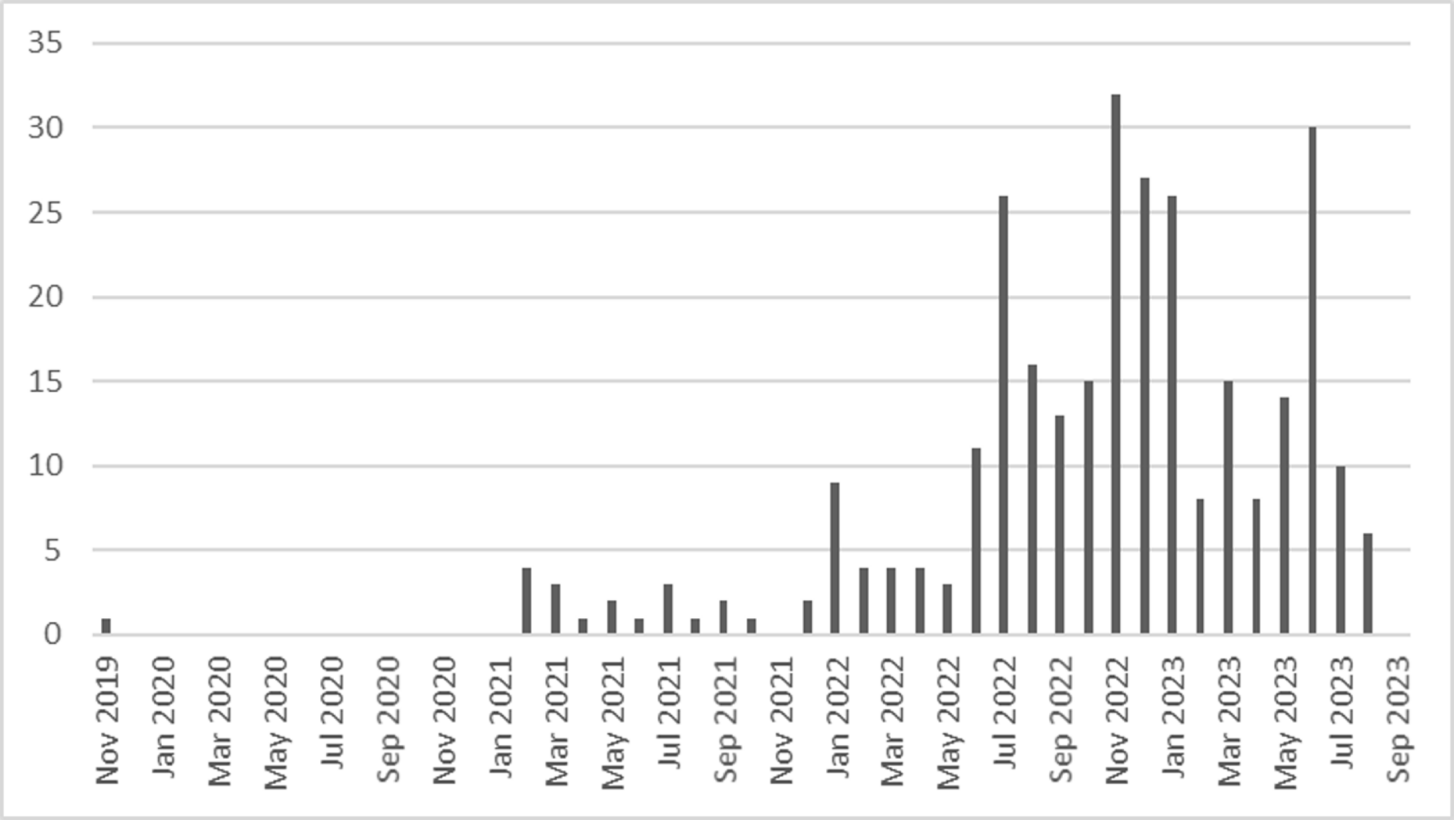
Frequency Distribution of Release Date (n =  302).

Videos coded as “99” were removed from the dataset for further analysis, as they were irrelevant to the purpose of the current study. To analyze the data and interpret the results in a convenient manner, videos coded as “0” were also removed, as they only occupied a small proportion of the videos (about 2%). After cleaning up these cases, a cleaned version of the dataset including 302 videos was formed. The oldest video in this dataset was released in November 2019. According to Fig 1, the number of videos related to foreign pseudohistory then rose in 2021 and became popular in 2022. Distributions of videos supporting and opposing foreign pseudohistory show no salient difference.

We further examined the central tendencies and dispersions of variables that represented the popularity of these videos, including numbers of views, comments, and bullet comments (a different kind of comment that moves across the screen like bullets while videos are playing) [47,48], likes, favorites, shares, and coins (a virtual currency in Bilibili that can be used to “tip” creators) rewarded to the videos. Results are shown in Table 2 below, including the popularities of the overall videos, videos supporting the claim of foreign pseudohistory, and videos opposing the claim of foreign pseudohistory, respectively. Medians are also reported as all six variables are heavily skewed. Overall, these descriptive data show that the videos opposing foreign pseudohistory received higher views, generated more comments and bullet comments, and were more popular (more likes, favorites, shares, and coins) than those videos promoting foreign pseudohistory.

**Table 2:** Central Tendency & Dispersion for Popularities (n_support_ =  213; n_oppose_ =  89).

Variable	Mean (Median)	Standard Deviation
View	45215.91 (21784.5)	62841.41
Support (1)	34621.39 (19003)	45739.31
Oppose (−1)	70571.33 (38903)	86865.5
Comment	1043.93 (696.5)	1159.31
Support (1)	851.38 (562)	964.98
Oppose (−1)	1504.76 (976)	1431.52
Bullet comment	202.59 (73.5)	449.13
Support (1)	166.17 (64)	432.19
Oppose (−1)	289.75 (114)	478.62
Like	2537.98 (1387.5)	4250.13
Support (1)	2414.09 (1395)	2911.7
Oppose (−1)	2832.47 (1318)	6421.49
Favorite	570.97 (321)	839.89
Support (1)	486.43 (304)	639.91
Oppose (−1)	773.29 (365)	1169.46
Share	229 (91)	521.29
Support (1)	170.61 (98)	259.59
Oppose (−1)	368.74 (84)	859.73
Coin	442.58 (146)	802.04
Support (1)	427.7 (145)	688.22
Oppose (−1)	478.18 (154)	1028.28

*Note*. Numbers are rounded to 2 decimal places.

A long-tail distribution was observed when counting the number of videos released by creators. The nine most prolific creators contributed 49% of the videos (*n* =  148), while the top nineteen creators in total accounted for 67.9% of the videos (*n* =  205). The top 19 instead of 20 creators were listed, as there were 17 creators who released two videos each, ranked right after the 19^th^ creator. Table 3 lists these creators as well as the number of videos they released correspondingly. The popularities of their videos are shown in Table 4. User IDs of these creators were replaced by randomized IDs to ensure privacy.

**Table 3:** Top 19 Creators.

Creator	Numbers of Videos in Dataset		
	Overall	Coded as “1”	Coded as “−1”
RID_1259	35	35	0
RID_1052	26	26	0
RID_1199	23	23	0
RID_1029	22	22	0
RID_1157	15	15	0
RID_1089	14	14	0
RID_1011	13	13	0
RID_1146	9	9	0
RID_1176	8	0	8
RID_1106	6	0	6
RID_1140	6	6	0
RID_1228	5	5	0
RID_1195	4	0	4
RID_1241	4	4	0
RID_1086	3	0	3
RID_1222	3	0	3
RID_1119	3	0	3
RID_1253	3	0	3
RID_1130	3	3	0
Overall	205	175	30

**Table 4:** Means of Popularity-Related Variables for Top 19 Creators’ Videos, Respectively.

Creator	View	Comment	Bullet	Like	Favorite	Share	Coin
RID_1259	40113	760	55	2613	632	213	177
RID_1052	39910	1348	319	2683	611	149	1519
RID_1199	23330	663	81	2139	375	166	197
RID_1029	45858	481	148	2388	364	126	295
RID_1157	13538	331	32	2569	229	42	81
RID_1089	26325	917	136	3792	742	208	517
RID_1011	22967	593	134	1280	354	190	340
RID_1146	20789	337	22	740	199	71	67
RID_1176	58592	2325	300	2143	707	225	901
RID_1106	21650	583	64	807	200	79	141
RID_1140	17850	734	52	1215	430	141	171
RID_1228	14142	702	47	1340	203	59	103
RID_1195	64978	1282	70	1913	492	138	80
RID_1241	20623	654	77	560	160	58	35
RID_1086	34976	406	139	2580	189	72	360
RID_1222	52572	639	106	1108	465	1264	167
RID_1119	44412	1511	91	841	247	40	87
RID_1253	71080	845	117	941	364	469	58
RID_1130	23330	831	72	845	268	55	131

*Note*. Numbers are rounded to integer.

As indicated by Table 3, most of the top creators took the position of supporting the claim of foreign pseudohistory, while a large portion of videos opposing the claim were produced by creators who were not on the list. It is noteworthy that prolific creators did not always create influential videos—only 4 of the 20 most viewed videos were produced by these most prolific creators. When looking at the overall numbers of videos supporting/opposing the claim of foreign pseudohistory, an average of 5.2 videos per author were uploaded among all videos supporting the claim, and the corresponding number was 1.53 for videos opposing the claim. In light of this, we may conclude that there was a relatively stable group of creators who continuously uploaded videos supporting the claim of foreign pseudohistory (regardless of the popularity of their videos), while the number of continuous creators who oppose the claim was limited.

### Popularities of videos

To further examine the second research question about the popularity of the videos, inferential statistics (e.g., t-tests) were used to explore if there were any significant differences between the supporting and opposing videos among the seven variables representing popularity (numbers of views, comments, bullet comments, likes, favorites, shares, and coins) in addition to descriptive data in Table 2. It should be noted that our dataset violated both the assumptions of normality as well as homoscedasticity for *t*-tests. Results from Shapiro-Wilk tests (to examine normality) and Levene’s tests (to examine homogeneity of variance) are reported in Table 5.

**Table 5:** Results of Assumption Tests.

Test	Group	Test Statistic						
		View	Comment	Bullet	Like	Favorite	Share	Coin
Shapiro-Wilk	Support	0.52***	0.67***	0.31***	0.62***	0.56***	0.51***	0.60***
	Oppose	0.66***	0.78***	0.58***	0.33***	0.56***	0.43***	0.43***
Levene’s		16.18***	9.60**	3.47	1.63	7.87**	10.97**	0.26

*Note*. ^* ^*p* < .05, ^**^p < .01, ^***^p < .001. Numbers are rounded to 2 decimal places.

In light of this, the current study used a combination of Trimmed Means *t*-tests (also called Yuen’s method) and Welch’s *t*-test, which provide a robust solution allowing for the presence of unequal variances as well as non-normal distributions [49]. For effect sizes, similarly, the frequently used Cohen’s *d* is not desirable, as it also requires homoscedasticity. Therefore, the current study applied explanatory measures of effect size as a robust approach instead, where values equal to 0.1, 0.3, and 0.5 correspond to small, medium, and large effect sizes, respectively [50]. Results are reported in Table 6.

**Table 6:** Results of Yuen-Welch t Tests.

	View	Comment	Bullet	Like	Favorite	Share	Coin
Test Statistic	3.73***	3.79***	3.12**	0.47	1.79	0.06	0.10
Degree of Freedom	58.76	66.08	65.31	85.95	63.44	72.69	109.39
Effect Size	0.51	0.46	0.32	0.06	0.19	0.03	0.05

*Note*. ^* ^*p* < .05, ^**^p < .01, ^***^p < .001. Trim =  0.2. Numbers are rounded to 2 decimal places.

As shown in the results, when referring to the numbers of views and comments (both normal and bullet ones), it can be concluded that videos opposing the claim of foreign pseudohistory were more popular (as shown in Table 2). However, when it comes to some other variables, including likes, favorites, shares, and coins, the differences between these two groups were no longer significant. Potential causes for these results will be discussed in a later section.

### Themes of videos

Among these 35 videos, there are 14 videos opposing foreign pseudohistory and 21 videos supporting foreign pseudohistory. Three major themes were identified from these videos, including: 1) how foreign civilizations and history were allegedly based on fabrication and plagiarism; 2) promoting foreign pseudohistory as a way to fight against Western-centrism; and 3) refuting and mocking foreign pseudohistory and enlightening the public about real history (please see Table 7 for details).

**Table 7:** Themes, descriptions, and sample quotations from data.

Themes	Descriptions	Sample Quotation (Typos and grammar errors were corrected by authors)
Pro-pseudohistory: Fabrication and plagiarism	How foreign civilizations and history were based on fabrication and plagiarism	Surprisingly, most Western people did not know where the techniques of printing presses came from until the 20^th^ century. They were careless and could not consider everything when they fabricated history... They intended to re-make Ancient Egypt as the origin of [Western] civilizations (video #118).
Pro-pseudohistory: Civil participation	Promoting pseudohistory as the way to participate in cultural and historical discourses	(We) must ensure all Chinese history academic community takes Chinese people’s benefits as the priority... Whoever cares about China and Chinese people, whoever has the discourse power (话语权) about [history]. We cannot give in and let Western scholars have the discourse power (video #109).
Opposing pseudohistory	Refuting and mocking pseudohistorical narratives or their believers	There are many ways to prove yourself while derogating others is not one of them (video #50). Was Ancient Egypt faked? A discussion on pseudohistory and historical nihilism (video #41). How do amateur historians invent pseudohistory to deny Chinese Civilization? [The universe of pseudohistory] (video #46)

The first theme emphasized fabricated foreign histories, civilizations, and historical relics. For example, according to video #118: “(The Colosseum) did not have a long history just like the Notre-Dame de Paris. The Notre-Dame de Paris only had a short history about 150–200 years instead of 800 years according to some evidence.”

According to this video, Western people forged ancient Egypt and ancient Sumer, serving as the foundations of Western civilizations. In another video (#370), the author concluded that it was the Chinese who first invented bicycles, steamboats, searchlights and heating lamps, primitive cameras, thermometers, and mechanical clocks. As noted in this video, “Western people did not have the basics of astronomical calendar, so they were not able to make mechanical clocks.”

The titles under this theme tended to use emotional appeals to argue that civilizations such as ancient Egypt, ancient Greece, and ancient Rome either did not exist or had shorter and simpler histories. For example, most of these titles tend to be: “Many of the histories of Western civilization written today are fabricated.” “Unveil and fight against the counterfeit Western pseudo-civilization in Kaifeng.” “Aristotle’s Complete Masterpiece has already been debunked. How many more books are actually forgeries?” “Pseudo-History in the West Explodes Along the Way (Part 4) Baseless, Unfounded Pseudo-Ancient Greece and Pseudo-Ancient Rome.”

For the second theme of these videos, many promoting pseudohistory argued that creating and promoting their opinions is the way to participate in cultural and historical discourses. According to [19], public discourses about scientific populism and conspiracy theories implied non-traditional forms of civic participation. Likewise, the popular debates about these foreign pseudohistory videos on Bilibili are indicators of Chinese media users’ non-traditional participatory acts to engage in cultural and historical discourses and challenge traditional academic elites. For example, one video (#190) argued:

“If those Chinese historians who studied how Western civilizations inspired Chinese civilization or Western learning influencing the East got promoted as professors or won awards, then Chinese history discipline will be dominated by Western-centrism and Chinese-worthless-ism (中国无价值论). How terrible it would be if that happened!”

Likewise, another video (#290) has a title calling “The ‘official science’ (官科 in original Chinese) often exhibits consistent arrogance and dominance.” This theme is consistent with previous findings on conspiracy theorists’ arguments that epistemic authorities are part of conspiracy regimes and that science communities should not be trusted [17,20,33].

Under the second theme, video creators supporting foreign pseudohistory were more concerned with promoting their group image and challenging Western-centrism regardless of the truthfulness of their arguments and evidence. As noted by Douglas [25], people support and promote conspiracies to restore and boost their group image threatened by outgroups. These videos argued that since foreign (especially Western) scholars could deny the existence of ancient Chinese dynasties, why not fight back by denying the existence of foreign civilizations. Many videos supporting foreign pseudohistory have titles such as “Cultural warfare in the Third World War, if Western pseudo-history dares to fight, China will accompany to the end” (video #244), which promote tension between Chinese and foreign civilizations, implying these video creators’ feelings of insecurity and desire to promote their ingroup image. One of these videos (#190) promoting pseudohistory justifies the legitimacy of foreign pseudohistory as the “ordinary people’s history” to challenge the Chinese academic elites’ voices and Western-centrism.

The third theme was used by those who opposed foreign pseudohistory to refute facts and mock either the arguments in these pseudohistorical narratives or the believers of pseudohistory themselves. For example, one of the videos (#1) opposing foreign pseudohistory argued that both foreign and Chinese archaeologists were questioning their own ancient artifacts and histories, and most of them were honest about their actual findings and later restorations and renovations. This video further argued that it is a common phenomenon for later generations to revise and add new things to their history. In other words, pseudohistory and forged history existed in both the West and China as part of normal human civilizations.

In response to those who promoted foreign pseudohistory to restore their group image threatened by Western-centrism, this video claimed such threat was overestimated and revealed that foreign scholars did not deny the existence of ancient Chinese civilizations at all. In addition, the same video also introduced European and U.S. American history textbooks in which most acknowledged the existence of the first Chinese ancient dynasty, Xia. That is to say, according to this video, many foreign pseudohistory supporters imagined a conspiracy theory about how Western-centrism denies Chinese history, civilization, and discourse power without any verified basis. Likewise, another video (#46) questioned the foreign-pseudohistory supporters’ fallacy that Western missionaries stole Chinese techniques to inspire the Western Renaissance. Furthermore, this video pointed out that since most historical events and achievements were interrelated somehow, promoting foreign pseudohistory to deny foreign history would finally result in Chinese pseudohistory denying China’s own history, techniques, and achievements.

Regarding some foreign pseudohistory supporters’ claims that ancient Egyptian pyramids were built by Europeans so the latter could forge ancient Egyptian civilization to serve as the basis of Western civilizations, another video (#50) systematically introduced how Egyptian pyramids were built by ancient Egyptians. This video further argued that we (Chinese) could question the existence of other civilizations rationally, while treating all foreign civilizations as fabricated makes no sense. As a result, these videos opposing foreign pseudohistory can be treated as non-traditional agents to facilitate and enlighten public discourse about real history.

The titles under this theme tended to use a sarcastic tone with counter-questions and usually mimicked the titles supporting pseudohistory in an exaggerated way. These titles include: “The pseudo-history theory is shameless” (video #158). “Are the Pyramids fake? Were they built by Europeans? What’s the real story?” (video #50) “Did Newton’s theory ‘plagiarize’ our achievements?” (video #8)

Overall, inductive thematic analysis of foreign pseudohistory videos identified several themes featuring video makers’ challenges to established historical knowledge. Foreign histories outside China were considered fake, inferior, and plagiarized based on Chinese classics. Other foreign-pseudohistory supporters consider promoting such pseudohistory a way to participate in cultural and historical discourses to boost Chinese discourse power and to battle against imagined Western-centrism threatening their ingroup image. In addition, established Chinese historical academic communities were questioned and attacked. Conspiratorial beliefs based on unverified speculations were promoted in these themes to intensify the relations between China and the rest of the world (specifically, the West) and discredit historical knowledge.

At the same time, there is also one important theme that was identified from these results as a counternarrative to refute conspiracies, enlighten the public, and mock such foreign pseudohistory via exaggerated and humorous mimicry. Videos opposing foreign pseudohistory also argued that the imagined threat from Western-centrism denying Chinese ancient civilization was most likely a speculation and Chinese discourse power could not be promoted by denigrating other civilizations.

## Discussions

*Videos opposing foreign pseudohistory have more viewers, but videos supporting foreign pseudohistory may also attract a considerable group of fans*. The overall number of pseudohistory videos has been increasing in the past few years, especially in 2022. The increase could be related to the frequent quarantine policies in China during that year, and the perceived blame and stigmatization from “the West” due to COVID-19 [51,52]. Quarantine policies reduced people’s offline activities so that they might focus on online issues [53], and the blame from outside might make people feel disrespected [51,52]. Based on the statistical descriptions and inferences presented in the previous sections, it can be concluded that, in general, there are more videos (in quantity) supporting the claim of foreign pseudohistory on Bilibili. However, those opposing the claim, in contrast, gained more attention from the public, which is reflected by the numbers of views and comments. In addition, many videos supporting the claim of foreign pseudohistory are posted by a small group of core creators, and still can attract a considerable group of viewers who are very willing to share the videos and support (“tip”) the creators. Although these videos supporting foreign pseudohistory have obtained less exposure compared with those videos opposing foreign pseudohistory, we did not observe significant differences in received likes, favorites, shares, and coins between these two types of videos.

Foreign pseudohistory has a subtle relationship with nationalism in developing countries. First, nationalism needs to be manifested in detailed themes instead of an over-simplified accusation. The manifested themes can be unreasonably glorifying one’s own history or downplaying other nations’ historical contributions. Second, foreign pseudohistory videos are sometimes related to distrusting intellectuals or the narration of “official science.” The tension between intellectuals and the general public cannot be simply explained by nationalism [54,55]. Third, the current study has found that at least among the highly-educated media users on Bilibili, the group criticizing foreign pseudohistory has a broader influence on the platform than the group supporting foreign pseudohistory (e.g., more view numbers, comments, and bullet comments). This finding indicates that even though nationalism may be increasing in the current world, rational people still make up a sizable group—at least among Bilibili users.

Theoretical Contributions. Theoretically speaking, the current study explored a new phenomenon of foreign pseudohistory and its popularity on a Chinese social media platform via a conspiracy perspective. The results from the thematic analysis yielded interesting patterns including unverified allegations questioning existing history scholarship and supportive arguments protecting established facts. Many videos supporting foreign pseudohistory showed distrust of historical authority. This finding adds to the understanding of conspiracy mentality, which is related to distrust in institutions and political cynicism [36,56]. Meanwhile, many videos opposing foreign pseudohistory also demonstrated the strength of rationality. The debate between supporting and opposing pseudohistory videos helped to depict a more complicated picture of developing countries’ societies. In addition, previous studies on conspiratorial thinking have suggested that believers of conspiracy theories tend to identify a scapegoat, such as Jews or big pharma, as a simple, immediate, and definitive answer instead of complicated and abstract social forces to address their problems [16,17]. Likewise, the pseudohistory believers in this study also identified Westerners’ plagiarism of Chinese civilization as a definitive answer to explain the development of foreign civilizations outside China.

According to [19], public discourse over conspiracy theories showed the public’s non-traditional civic engagement. Hence, these foreign pseudohistory videos (regardless of their positions) on Bilibili and the discussions over them can be considered non-traditional forms of civic participation in cultural and historical discourse. It should be noted that such civic participation can result in both conspiracies and enlightenment about how Chinese view foreign and their own civilizations. Thus, the current study provided empirical evidence from Chinese social media platforms to support previous conspiracy-related studies and their effect on public discourses, which also extends the scope of conspiracy literature to the Chinese Internet context.

In summary, the current study yielded implications for future scholarship in nationalism, conspiracy theory, and non-traditional online civic participation in the Chinese context. This is the first empirical study adopting multiple methods to explore an underexamined field regarding online conspiracy messages in a developing country such as China. The differences between videos supporting and opposing foreign pseudohistory also suggested the heterogeneity of Chinese cyberspace, which is worthwhile for future examination.

## Limitations

A limitation of the current study is the dataset, which does not allow more inference on the causality between video patterns/themes and video popularity. The current study did not track longitudinal data of videos, so it can only identify the differences in video popularity between two positions of videos, but cannot conclude that the popularity is caused by different positions. Researchers plan to conduct experimental studies and track longitudinal data of videos in the future to examine the causal relationship between video positions/themes and video popularity.

The current study adopted multiple methods to examine foreign pseudohistory videos on a Chinese social media platform, Bilibili. Future studies might consider exploring foreign pseudohistory videos on other Chinese social media platforms to study whether there are any platform-based differences in terms of themes and popularity of these videos. In addition, this study only analyzed the content of videos, the themes of titles, and the user responses such as sharing and viewing numbers of the 302 videos posted before September 2^nd^, 2023. Some details such as the user-generated comments and real-time bullet comments [47,48] from these videos need to be examined in the future. Although content analysis was conducted to detect whether these videos support pseudohistory or not, it is theoretically interesting to examine whether the comments support the content of these videos. Besides, future studies might examine if there are any social bots [57] posting comments under these videos.

Another aspect the current study has not yet addressed is the dynamics and network structures among the audiences of these videos. As mentioned above, videos supporting the claim may have a smaller but more cohesive group of fans. It would be valuable if future studies could identify this group of audiences and examine their behavioral patterns. Besides, we do not yet know to what extent these two groups of audiences, supporting and opposing the claim of foreign pseudohistory, engage in a “debate,” or whether they are two distanced groups with few intergroup interactions. If the latter situation is true, it means that so far the effort of persuading the believers of foreign pseudohistory to alter their beliefs has not reached the target audience, as they are living in their filter bubbles. Future studies may use other techniques, like network analysis, to address these remaining questions.

## Conclusion

The current study has conducted an exploratory analysis of foreign pseudohistory on Chinese video websites. By collecting information from 302 related videos on Bilibili, the study has found that videos opposing foreign pseudohistory have more viewers, but videos supporting foreign pseudohistory may also attract a considerable group of fans. The thematic analysis identified different themes from these videos about why video creators promote or oppose foreign pseudohistory, such as countering Western-centrism (for the former) or enlightening the public about real history (for the latter).

To sum up, we find that on one side, foreign pseudohistory has a subtle relationship with nationalism and distrust toward intellectuals. On the other side, there are more videos opposing foreign pseudohistory than expected, which shows a group of rational people who are against the spread of conspiracy theories in culture. This finding indicates that - unlike some previous literature has suggested [7,17,31]—it is still too early to conclude the current world is overwhelmed by irrational behaviors, and rational people still make up a sizable group. This idea is also supported by misinformation studies focusing on social news, in which scholars find that misinformation is overestimated online and consuming misinformation does not equal believing it [58].

In addition, the current study has concluded these findings in the Chinese context. However, considering other developing countries may experience similar phenomena of culture and history-related conspiracy theories (e.g., Russia in the 1990s and South Korea in the 1970s), the findings could encourage further studies focusing on other developing countries and explore their motivations and narration patterns of culture and history-related conspiracy theories.

## Supporting information

S1 File Video List 101423. (XLSX)
